# Diplomatic roles of regional coordinators for WHO Member States

**DOI:** 10.2471/BLT.25.294847

**Published:** 2026-04-16

**Authors:** Nikica Daraboš, Monica Stanovic, Danira Matijaca

**Affiliations:** a University North, Ulica 104. brigade 3, 42000 Varaždin, Croatia.; b Mailman School of Public Health, Columbia University, New York, NY, United States of America.; c School of Medicine, University of Split, Split, Croatia.

## Abstract

**Objective:**

To examine the coordination functions, decision-making processes and consensus-building strategies of World Health Organization (WHO) regional coordinators of Member States in Geneva within the WHO’s federal governance structure.

**Methods:**

In this qualitative study, we interviewed 12 current and former regional coordinators from five WHO regions (African, the Americas, South-East Asia, European and Eastern Mediterranean) using a semi-structured questionnaire. A regional coordinator from the Western Pacific Region later provided a written response to the questionnaire which we report separately due to methodological and time differences. We analysed the data using a hybrid thematic approach combining initial coding assisted by artificial intelligence with our rigorous interpretive validation.

**Findings:**

We identified eight main coordination functions. Core liaison and information-sharing were functions undertaken by regional coordinators in all the regions, while position development and meeting organization were undertaken by regional coordinators in most regions. Coordination complexity and consensus-building strategies, such as bilateral mediation and issue reframing, varied depending on regional political dynamics and institutional capacities. A formal job description and procedural handbooks were lacking, and therefore the regional coordinator role was a negotiated practice dependent on informal knowledge transfer.

**Conclusion:**

Regional coordinators serve as boundary-spanners linking regional autonomous governance with global health governance. To address the absence of documented institutional knowledge, we propose developing formal induction curricula, cross-regional mentorship pairing and harmonized procedural handbooks. These measures will strengthen regional voices and ensure more inclusive participation in global health diplomacy.

## Résumé

**Objectif:**

Examiner les fonctions de coordination, les processus décisionnels et les stratégies de recherche de consensus des coordinateurs régionaux des États membres de l’Organisation mondiale de la Santé (OMS) à Genève, dans le cadre de la structure de gouvernance fédérale de l’OMS.

**Méthodes:**

Dans le cadre de cette étude qualitative, nous avons interrogé 12 coordinateurs régionaux actuels et anciens issus de cinq régions de l’OMS (Afrique, Amériques, Asie du Sud-Est, Europe et Méditerranée orientale) en leur soumettant un questionnaire semi-structuré. Un coordinateur régional de la région du Pacifique occidental a par la suite fourni une réponse écrite au questionnaire, que nous présentons séparément en raison de différences méthodologiques et de calendrier. Nous avons analysé les données à l’aide d’une approche thématique hybride combinant un codage initial assisté par l’intelligence artificielle et notre validation interprétative rigoureuse.

**Résultats:**

Nous avons identifié huit fonctions de coordination principales. La liaison de base et le partage d’informations étaient des fonctions assumées par les coordinateurs régionaux dans toutes les régions, tandis que l’élaboration de positions et l’organisation de réunions étaient assurées par les coordinateurs régionaux dans la plupart des régions. La complexité de la coordination et les stratégies de recherche de consensus, telles que la médiation bilatérale et le recadrage des enjeux, variaient selon des dynamiques politiques régionales et des capacités institutionnelles. En l’absence de description formelle du poste et de manuels de procédures, le rôle de coordinateur régional est une pratique négociée reposant sur un transfert informel de connaissances.

**Conclusion:**

Les coordinateurs régionaux assurent un rôle d’intermédiaire culturel entre la gouvernance régionale autonome et la gouvernance mondiale en matière de santé. Pour pallier l’absence de connaissances institutionnelles documentées, il convient de mettre en place des programmes formels de formation initiale, des binômes interrégionaux de mentorat et des manuels de procédures harmonisés. Ces mesures renforceront la voix des différentes régions et garantiront une participation plus inclusive à la diplomatie mondiale en matière de santé.

## Resumen

**Objetivo:**

Examinar las funciones de coordinación, los procesos de toma de decisiones y las estrategias de construcción de consensos de los coordinadores regionales de los Estados Miembros de la Organización Mundial de la Salud (OMS) en Ginebra, en el marco de la estructura federal de gobernanza de la OMS.

**Métodos:**

En este estudio cualitativo, se entrevistó a 12 coordinadores regionales actuales y anteriores de cinco regiones de la OMS (África, las Américas, Asia Sudoriental, Europa y el Mediterráneo Oriental) mediante un cuestionario semiestructurado. Un coordinador regional de la Región del Pacífico Occidental proporcionó posteriormente una respuesta escrita al cuestionario, que se presenta por separado debido a diferencias metodológicas y temporales. Los datos se analizaron mediante un enfoque temático híbrido que combinó una codificación inicial asistida por inteligencia artificial con una validación interpretativa rigurosa.

**Resultados:**

Se identificaron ocho funciones principales de coordinación. Las funciones de enlace y de intercambio de información se llevaron a cabo en todas las regiones, mientras que el desarrollo de posiciones y la organización de reuniones se realizaron en la mayoría de ellas. La complejidad de la coordinación y las estrategias de construcción de consensos, como la mediación bilateral y la reformulación de cuestiones, variaron en función de las dinámicas políticas regionales y de las capacidades institucionales. Se constató la ausencia de una descripción formal del puesto y de manuales de procedimientos, por lo que el rol del coordinador regional constituyó una práctica negociada dependiente de la transferencia informal de conocimientos.

**Conclusión:**

Los coordinadores regionales actúan como elementos de enlace que conectan la gobernanza regional autónoma con la gobernanza mundial de la salud. Para abordar la ausencia de conocimiento institucional documentado, se deberían desarrollar programas formales de incorporación, sistemas de mentoría interregional y manuales de procedimientos armonizados. Estas medidas fortalecerán las voces regionales y asegurarán una participación más inclusiva en la diplomacia de la salud mundial.

## ملخص

**الغرض:**

فحص وظائف التنسيق، وعمليات صنع القرار، واستراتيجيات بناء التوافق لدى المنسقين الإقليميين للدول الأعضاء في الهيكل الإداري الاتحادي لمنظمة الصحة العالمية (WHO) في جنيف.

**الطريقة:**

قمنا في هذه الدراسة النوعية بإجراء مقابلات مع 12 منسقًا إقليميًا حاليًا وسابقًا من خمسة أقاليم تابعة لمنظمة الصحة العالمية (أفريقيا، والأمريكتان، وجنوب شرق آسيا، وأوروبا، وشرق المتوسط) باستخدام استبيان منظم جزئيًا. قام أحد المنسقين الإقليميين من إقليم غرب المحيط الهادئ لاحقًا بتقديم رد مكتوب على الاستبيان، والذي نعرضه بشكل منفصل نظرًا للاختلافات المنهجية والزمنية. وقمنا بتحليل البيانات باستخدام أسلوب موضوعي مختلط يجمع بين الترميز المبدئي المدعوم بالذكاء الاصطناعي، والتحقق التفسيري الدقيق الذي أجريناه.

**النتائج:**

قمنا بتحديد ثماني وظائف تنسيق رئيسية. كان كل من الاتصال الأساسي وتبادل المعلومات من الوظائف التي اضطلع بها المنسقون الإقليميون في جميع الأقاليم، بينما تولى المنسقون الإقليميون في معظم الأقاليم مسؤولية تطوير المواقف وتنظيم الاجتماعات. تباينت تعقيدات التنسيق واستراتيجيات بناء التوافق، كالوساطة الثنائية وإعادة صياغة القضايا، وذلك تبعًا للديناميكيات السياسية الإقليمية والقدرات المؤسسية. كان هناك نقص في الوصف الوظيفي الرسمي والكتيبات الإجرائية، ولذلك كان دور المنسق الإقليمي هو ممارسة تفاوضية تعتمد على نقل المعرفة غير الرسمي.

**الاستنتاج:**

يعمل المنسقون الإقليميون كحلقة وصل بين الحوكمة الذاتية الإقليمية وحوكمة الصحة العالمية. وللتعامل مع غياب المعرفة المؤسسية الموثقة، ينبغي تطوير مناهج تعريفية رسمية، وتنسيق برامج الإرشاد بين المناطق، ووضع كتيبات إجرائية موحدة. ستعزز هذه التدابير الأصوات الإقليمية وتضمن مشاركة أشمل في دبلوماسية الصحة العالمية.

## 摘要

**目的:**

旨在考察世卫组织 (WHO) 成员国在日内瓦的区域协调员在世卫组织 (WHO) 联邦治理结构下的协调职能、决策流程及共识构建策略。

**方法:**

在本次定性研究中，我们使用半结构化问卷，访问了来自世卫组织五大区域（非洲、美洲、东南亚、欧洲和东地中海）的 12 位现任及前任区域协调员。随后，一位西太平洋区的区域协调员也对问卷进行了书面回答，由于方法论和时间不同，我们将进行单独汇报。我们使用混合主题方法，结合借助人工智能辅助的初始编码和我们严谨的解释性验证，对数据进行了分析。

**结果:**

我们确定了八大主要协调职能。核心联络和信息共享是所有区域的区域协调员承担的职能，而大部分区域的区域协调员还承担立场制定和会议组织的职能。协调复杂度和共识构建策略，例如双边斡旋和议题重构，则根据区域政治动态和机构能力不同而有所不同。因为没有正式的职位描述和程序手册，所以区域协调员职能的实际操作主要依靠非正式的知识传递进行协调。

**结论:**

区域协调员主要担任边界跨越者的角色，连接区域自主治理和全球卫生治理。应制定正式的入职培训课程、跨区域导师配对和统一的程序手册，以解决缺少制度化知识文件的问题。这些办法将增强区域发言权，确保各方能以更加包容性的方式参与全球卫生外交。

## Резюме

**Цель:**

Изучить координационные функции, процессы принятия решений и стратегии достижения консенсуса региональных координаторов государств-членов Всемирной организации здравоохранения (ВОЗ) в Женеве в рамках федеративной структуры управления ВОЗ.

**Методы:**

В рамках данного качественного исследования авторы опросили 12 действующих и бывших региональных координаторов из пяти регионов ВОЗ (Африканского, Американского, стран Юго-Восточной Азии, Европейского и Восточного Средиземноморья), используя полуструктурированный опросный лист. Координатор, представляющий регион западной части Тихого океана, позднее предоставил письменные ответы на вопросы. Эти ответы мы приводим отдельно в связи с различиями в методологии и сроках. Данные были проанализированы с использованием гибридного тематического подхода, сочетающего первоначальное кодирование с применением искусственного интеллекта при условии строгой интерпретационной валидации.

**Результаты:**

Было выявлено восемь основных координационных функций. Взаимодействие и обмен информацией были основными функциями для региональных координаторов во всех регионах, в то время как выработка позиций и организация встреч – в большей части регионов. Сложность координации и стратегии достижения консенсуса, такие как двустороннее посредничество и переформулирование вопросов, различались в зависимости от региональной политической динамики и институционального потенциала. Ввиду отсутствия официальных должностных инструкций и процедурных руководств роль регионального координатора на практике определялась постоянно меняющимися договоренностями и зависела от неформальной передачи знаний.

**Вывод:**

Региональные координаторы выполняют функцию связующего звена между автономным региональным управлением и глобальным управлением в сфере здравоохранения. Для устранения проблемы отсутствия документированных институциональных знаний следует разработать официальные программы введения в должность, механизмы межрегионального наставничества и унифицированные процедурные руководства. Эти меры усилят голос регионов и обеспечат более инклюзивное участие в глобальной дипломатии в сфере здравоохранения.

## Introduction

The World Health Organization (WHO) operates through a distinctive dual governance structure among United Nations (UN) agencies.^1^^,^^2^ The World Health Assembly (WHA), comprising all 194 Member States of WHO, is WHO’s supreme decision-making body which meets annually in Geneva, and the Executive Board, with 34 Member States, is WHO’s executive body.^1^ At the regional level, six regional committees oversee regional offices for Africa, the Americas, South-East Asia, Europe, the Eastern Mediterranean and the Western Pacific.^3^

When WHO was established in 1948, integrating the existing Pan American Health Organization (PAHO) necessitated a federalistic structure, with the regional directors being selected directly by the Member States in the respective regions.^1^ Unlike other UN agencies where regional structures have mainly administrative functions,^1^ WHO’s regional committees are genuine decision-making bodies, with considerable autonomy to adapt global policies to regional contexts.^4^^,^^5^

This federalistic design creates complex coordination requirements. Regional committee resolutions inform WHA agendas, while global policies require regional-level adaptation.^3^^,^^5^^–^^9^ Therefore, consensus must be built both within and across regions.^10^^–^^16^ WHO regional coordinators of Member States in Geneva, appointed by the Member States of the region, operate as Member State representatives facilitating this coordination. Unlike WHO staff members or traditional diplomats, regional coordinators are boundary-spanners who bridge organizational levels, facilitate information flows and coordinate regional positions during negotiations.^17^^–^^22^

Despite their importance, research on regional coordinators' roles remains limited. Global health governance literature has focused mainly on WHO headquarters’ decision-making or regional office operations, overlooking coordination mechanisms linking these levels.^5^^,^^23^^,^^24^ Studies have examined tensions between headquarters and regions^4^^,^^5^^,^^25^ and Member State negotiations.^26^^–^^29^ Understanding how coordination functions in WHO’s federalistic structure is essential for comprehending global health governance in practice.^30^^–^^32^

We used three complementary theoretical approaches. Boundary-spanning theory conceptualizes regional coordinators as actors who link organizational subsystems, process external information and represent their regional offices externally. Information processing and external representation are the main functions.^17^^,^^18^^,^^33^^–^^39^ Policy coordination theory, particularly the coordination spectrum, provides analytical tools for understanding the degree of coordination across governance levels.^30^ Global health diplomacy scholarship situates regional coordinator activities within broader multilateral negotiation processes.^26^^,^^27^^,^^40^^,^^41^

Our qualitative study asks three questions. What coordination functions do regional coordinators perform? How do regional coordinators navigate decision-making within WHO’s dual governance structure? What consensus-building strategies bridge regional and global levels?

## Methods

We used in-depth, semi-structured interviews to collect information on the experiences, perspectives and opinions of current and past regional coordinators on their coordination practices, communication methods, training and guidance recommendations, and required skills and competencies. 

### Participants

We used purposive sampling and snowball sampling techniques to identify and contact regional coordinators. We interviewed regional coordinators who agreed to participate and also asked them to refer us to other current or former coordinators within their region. We conducted 12 in-depth interviews with current or former regional coordinators, representing five WHO regions: the African Region, Region of the Americas (PAHO), South-East Asia Region, European Region and Eastern Mediterranean Region. No participants from the Western Pacific Region agreed to participate under our data collection conditions (audio-recorded Zoom interviews). Subsequently, a regional coordinator from this region provided written responses to the interview questions 10 months after primary data collection. These data are presented separately and were not integrated into the primary thematic analysis. The regional coordinators had different professional backgrounds: five had a medical or public health education and four had diplomatic or foreign affairs experience. Most regional coordinators (11) had standard annual appointments, and rotation in alphabetical order by country name was the main selection mechanism, reported by seven participants.

### Data collection and analysis

We conducted semi-structured interviews via Zoom between March and April 2025. Interviews lasted 45–60 minutes. We recorded and transcribed the interviews using Clipto.ai (Clipto, Inc., Palo Alto, United States of America). We sent the transcripts to the participants for transparency and review. Data confidentiality was maintained through secure storage of recordings and transcripts, with access restricted to the research team, and by not attributing quotes to named individuals in the analysis. 

We used a hybrid approach for data analysis, combining initial coding assisted by artificial intelligence (AI) with rigorous human interpretive analysis. We initially processed transcripts using Claude (Anthropic, San Franciso, USA) to systematically identify recurring topics and preliminary thematic categories in the 12 interviews. To validate AI-generated codes, we independently reviewed codes and identified where AI categorization missed contextual nuances. We iteratively refined codes through consensus and discussion, and the final thematic framework represents our consensus based on the participants’ accounts.

To ensure the relevance of themes, we assigned a frequency weight to each identified theme to reflect the number of participants who independently raised it. We classified themes as universal (raised by all 12 participants), very high (10 or 11 participants), high (eight or nine participants), moderate (five to seven participants) or emergent (three or four participants). This approach differentiates between core cross-regional functions and context-specific observations, thereby strengthening analytical robustness beyond purely descriptive categorization.

### Coordination complexity assessment

We characterized coordination complexity based on indicators that emerged from participants’ answers, namely: organizational scale (number of Member States requiring coordination); structural coordination mechanisms (presence and development of formal coordination structures); political administrative context (diversity in governance systems, political tensions and diversity in health-system capacities); and coordination frequency and formality (reported complexity and regularity of coordination activities). This characterization reflects participants’ own descriptions and should be understood as a qualitative framework rather than a quantitative measure. 

### Ethics

The Research Ethics Committee at the University North (Sveuciliste Sjever), Croatia, approved the study protocol. We gave participants an informed consent form before participation, which explained that they could withdraw from the study at any stage. All participants gave their written informed consent before the interviews and anonymity was maintained.

## Results

### Coordination

Eight main coordination functions emerged for regional coordinators, with varying frequencies and regional adaptations (Table 1). Core liaison and information-sharing were universal functions, while position development and meeting organization were noted by almost all participants (11/12 and 10/12, respectively). Functions with moderate frequency (representation, nomination management and capacity-building) appeared more context-dependent.

**Table 1:** Functions and responsibilities WHO regional coordinators

Function	Description	Frequency^a^	Key activities	Regional variations
Core liaison	Acting as an intermediary between WHO, regional offices, and Member States in Geneva	Universal (12/12)	WHO–Member State liaison; regional office bridge; Geneva–national ministries communication; cross-regional communication	Performed by all regions with varying institutional support levels
Information-sharing	Facilitating systematic communication and information distribution	Universal (12/12)	Meeting updates; process notifications; emergency communication; document circulation	Range in methods from formal emails to groups in messaging apps depending on regional culture
Position development	Building coordinated regional positions and consensus	Very high (11/12)	Statement preparation; consensus-building; position mapping; joint negotiation positions	African Region’s troika system; Region of the Americas’ support from PAHO; European Region’s management of political tensions
Meeting organization	Managing various types of coordination meetings	Very high (10/12)	Pre-event meetings; side meetings during World Health Assembly sessions; briefing organization; cross-regional meetings	South-East Asia Region’s preferred informal canteen meetings; African Region’s use of formal meetings in conference settings
Representation	Advocating for regional interests in various forums	High (8/12)	Standing Committee of the Regional Committee; Executive Board Bureau; Director-General meetings; negotiations to provide a regional voice	Unique European Standing Committee reporting; quarterly meeting of the African Region’s Regional General; integration of the Region of the Americas and PAHO
Nomination management	Managing regional representation in WHO bodies	Moderate (7/12)	Intergovernmental Negotiating Body Bureau consultations; selected working group nominations; representative selection; bureau transitions	More common in larger regions (African and European regions) with more seats to fill across WHO governing bodies
Capacity-building	Supporting Member States with constrained resources	Moderate (6/12)	Small mission inclusion; procedural guidance; training facilitation; new attaché mentoring	Important in the African and South-East Asia regions due to capacity constraints; less needed in the European Region
Crisis response	Coordinating during health emergencies and crises	Variable	Pandemic response; natural disaster coordination; emergency communication	Variation in crisis types and coordination complexity by regional context and Member State capacities

App: application; PAHO: Pan American Health Organization; WHO: World Health Organization.

^a^ Frequency classifications reflect the number of participants (12) who independently raised each function during thematic analysis: universal: 12; very high: 10–11; high: 8–9; moderate: 5–7; emergent: 3–4. Functions referenced by fewer than three participants are reported as individual contextual observations rather than thematic findings.

Coordination approaches varied considerably across regions based on political dynamics and institutional capacity (Table 2). The African Region has a sophisticated system (including an African Union-based troika arrangement of the current, outgoing and incoming regional coordinator) for the regional coordinator to manage its 47 Member States. The Region of the Americas benefits from the institutional support of PAHO, creating a triangular coordination mechanism among Member States, PAHO and WHO headquarters. Coordination in the South-East Asia Region operates through informal consensus processes. Coordination in the European Region has distinct operational challenges because of political tensions and European Union membership.

**Table 2:** Regional coordination complexity

WHO region	Countries	Complexity level	Distinctive features	Coordination challenges
Africa	47	High	Troika system; African Union integration; cross-hub coordination	Capacity constraints; development diversity
Europe	53	High	Reporting by the Standing Committee of the Regional Committee; managing political tensions between Member States	Political tensions between Member States; European Union versus non-European Union dynamics
Americas	35	Medium	Integration with PAHO; formal procedures	Economic diversity and political diversity among Member States
Eastern Mediterranean	22	Medium	Written guidelines; cultural sensitivity	Political instability; religious considerations
South-East Asia	11^a^	Lower	Informal approaches; small size	Limited capacity; single health attaché covering multiple portfolios

PAHO: Pan American Health Organization; WHO: World Health Organization.

^a^ At the time of the study, Indonesia was assigned as a Member State of WHO South-East Asia Region.

### Regional consensus-building

Coordination in the African Region and South-East Asia Region showed the strongest commitment to consensus, with one regional coordinator emphasizing, “The good thing about our region is we really support consensus building. So, we don’t usually go for votes.” However, when consensus is not reached, a fallback applies. “In cases where we could not agree, we do not vote, but each country then speaks in their own national capacity.”

The Region of the Americas uses pragmatic flexibility, acknowledging the region’s diversity. “We’re not all the same, even though we are in the western hemisphere.” The European Region has adopted structured procedural approaches in response to political tensions, with coordinators noting how Member State tensions complicate regional statements. When divergent positions arise, coordinators allow countries to speak in a national capacity rather than pursuing unified positions.

Decision-making mechanisms range from informal consultation to systematic polling. In smaller regions, personal relationships are key. A former South-East Asia Region coordinator noted, “If we cannot reach consensus, then we do not proceed.” Coordinators from the Region of the Americas noted their use of WhatsApp (Meta, Menlo Park, USA) polling for routine logistical decisions.

While the African Region and South-East Asia Region have a strong commitment to full agreement, the European Region’s political tensions have made joint regional statements effectively unachievable in some instances. Capacity constraints compound these challenges: many smaller missions lack dedicated health attachés, reinforcing asymmetries between well-resourced and resource-constrained delegations.

### Tension resolution

Regional coordinators use tension resolution strategies with different levels of effectiveness across regions (Table 3). Bilateral mediation is the most commonly used strategy and preferred for disagreement resolution, with coordinators emphasizing individual consultation before multilateral meetings. The Eastern Mediterranean Region coordinators described systematic approaches when dealing with tensions. “I had to specifically address certain issues, and I had to perhaps call certain colleagues in informal meetings, try to understand their concerns, and try to deal with the situation offline.”

**Table 3:** Tension resolution strategies used by WHO regional coordinators and strategy effectiveness

Strategy type	No. of coordinators using the strategy (*n* = 12)	Regional preference	Reported effectiveness	Resource requirements	Skills required
Bilateral mediation	10	All regions	High for personal tensions	Low (coordinator time)	Interpersonal skills; cultural sensitivity
Issue reframing	8	Americas and Eastern Mediterranean most frequently	Medium for substantial disagreements	Medium (analytical capability)	Policy knowledge; creative thinking
Partial consensus	7	African, Americas and South-East Asia	High for process maintenance	Low (flexibility)	Diplomatic compromise skills
Offline consultation	9	All regions	High for sensitive issues	Medium (time, access)	Confidential relationship management
External mediation	3	African (African Union) and Americas (PAHO)	Variable	High (institutional support)	Institutional relationships

PAHO: Pan American Health Organization; WHO: World Health Organization.

Reframing issues emerged as a strategy for managing significant disagreements without requiring position changes. In the Region of the Americas, rather than trying to resolve all disagreements, coordinators emphasize areas of agreement while acknowledging differences.

Regional statement development is the most visible coordination output. Two main models emerged. First, coordinator-initiated statements, where coordinators took the initiative, as noted by one coordinator, “Sometimes when you take [the] initiative, others will adopt it because they might be embarrassed to say no, since they didn’t make the effort themselves.” Second, ministry-initiated statement development, where coordination occurs through officials in national health ministries. This strategy is used widely in the African Region.

### Supplementary perspective

While data from the Western Pacific Region was not integrated into the thematic analysis due to methodological and time differences, the later response of a regional coordinator provided key observations on coordination approaches used in this region.

The role of the regional coordinator is narrower than accounts from other regions and is focused mainly on informally eliciting Member State views, identifying nominations for elected positions within WHO bodies and conveying information to Geneva-based missions. Unlike coordinators from other regions, the coordinator of the Western Pacific Region reported no involvement in regional committee coordination or development of policy positions. The coordinator characterized the region’s approach as different from developing unified statements, noting that regional diversity means, “the region doesn’t aim for unified statements on most issues,” with regional statements to governing bodies described as infrequent. Communication occurs mainly by email, mostly among technical staff rather than heads of mission. Unlike rotation systems reported elsewhere, the same Member State has served as coordinator for the Western Pacific Region for 5 years, with “no written or commonly understood procedures” for selection.

## Discussion

We explored the work of the WHO Member State regional coordinators in Geneva, the challenges they face and the strategies they use. All regional coordinators perform core liaison and information-exchange functions. The most politically sensitive but common function among the coordinators is developing a common position at the regional level. Reaching consensus requires strong negotiation and diplomatic skills, especially in heterogeneous political environments.

Unlike WHO regional coordinators who operate within constitutionally mandated regional governance structures with policy-making authority, coordination in other organizations serves mainly administrative and logistical rather than substantive policy functions. For example, in the World Trade Organization, regional groupings and developing country coalitions are coordinated through rotating coordinators selected based on Member States’ willingness and capacity. Their role focuses on tactical negotiation support during ministerial conferences rather than ongoing policy coordination.^42^ Similarly, coordination of regional groups of the UN General Assembly is mostly for procedural purposes such as Security Council seat allocation and voting coordination, with chairs rotating monthly among Member States.^43^ These coordinators lack the authority to formulate policy which WHO regional coordinators have, and they do not engage in substantive development of positions, capacity-building or crisis coordination, which we found WHO regional coordinators did. Unlike them, WHO regional coordinators participate in developing region-specific health policies, making their role substantially more complex and politically sensitive.

A striking finding is that no formal job description, procedural handbook or institutional guidance exists for the regional coordinator role. One coordinator noted that from the start there were no rules or a handbook to outline what the role of coordinator was. This lack of a formal role definition has significant implications for how coordination functions in practice. First, role construction is negotiated rather than prescribed. Each coordinator interprets and enacts the role based on regional context, personal background and institutional relationships, which creates considerable variation in how the position functions across regions. Second, institutional knowledge remains fragile and is dependent on informal knowledge transfer between coordinators rather than codified procedures. As coordinators reported, guidance came from one colleague or similar informal mentorship rather than systematic onboarding. Third, accountability mechanisms are ambiguous. Without formal expectations, assessing coordinator performance or providing targeted support becomes challenging. Finally, this flexibility enables context-appropriate innovation but limits systematic transfer of effective practices between regions. This finding challenges assumptions about institutionalization in multilateral organizations and reveals tensions between formalization and flexibility in coordination mechanisms within federalistic governance structures. The absence of codified procedures is not merely an oversight but reflects deeper questions about how informal diplomatic roles function within formal organizational hierarchies.

Our findings show how regional coordinators navigate complex power dynamics and authority relationships. Regional coordinators at the intersection of competing frameworks, simultaneously represent national interests, uphold WHO public health norms and build regional consensus. In contexts characterized by significant capacity differences between Member States, regional coordinators perform bridging functions, ensuring smaller states with limited Geneva representation can participate meaningfully in coordination processes. Some Member States lack permanent health representatives, requiring additional outreach efforts to avoid inadvertently marginalizing resource-constrained states. When Member States hold different positions on sensitive issues, coordinators develop sophisticated mechanisms to maintain coordination channels. Coordinators emphasized the importance of personal relationships and diplomatic skill in navigating politically sensitive coordination challenges while maintaining regional cohesion. These practices show how coordination mechanisms can either sustain or mitigate existing power inequalities, with structured approaches including capacity-building functions working to counterbalance resource disparities.

Our findings also show how regional blocs adapt coordination mechanisms to current governance realities while building on historical patterns. Coordinators described shifts from mainly procedural coordination towards substantive policy development functions, reflecting broader trends where regional voices increasingly shape global agenda-setting and development of norms. The complexity of coordination mechanisms varies across regions based on institutional maturity, with some regions having formalized structures developed over decades (the African Region’s troika system and the Region of the Americas' integration with PAHO), while others maintain flexible informal approaches adapted to smaller membership or different political cultures. This diversification represents adaptive responses to distinct regional contexts rather than a single evolutionary pathway. Contemporary challenges, such as managing political tensions between Member States, ensuring inclusive participation despite differences in capacity, and balancing regional autonomy with global coherence, reflect long-standing tensions in WHO’s federalistic structure taking new forms. 

In addition, the growing need for diplomatic and negotiation skills in conducting regional coordination duties underscores the possible need for formal training before starting the regional coordinator role. As concluded from our study, the role of regional coordinators goes beyond technical coordination. The role requires working within complex interdisciplinary structures, where disagreement resolution, understanding of the geopolitical context and preservation of neutrality are crucial. Regional coordinators also highlighted the need for stronger institutional support, especially in regions where small Member States have limited resources and often lack sufficient resources or permanent health representatives in international bodies.

Decision-making mechanisms vary from informal discussions and digital surveys to formal procedures. Digital tools can facilitate and speed up decision-making, but raise questions about the legitimacy of the process. Our respondents noted that bilateral discussions before formal meetings or in the case of dispute resolution help reduce tensions and prepare the ground for meetings and regional consensus. A strong indicator of successful coordination at a regional level is the development of joint statements. At times, the initiative to improve regional coordination comes from national health ministries in Member States, with health ministries drafting statements. At other times, the initiative to draft regional statements comes proactively from regional coordinators. Despite the efforts to improve coordination and produce joint statements, political tensions can sometimes undermine the efforts to achieve a unified position, which only reduces the visibility of the regional actors in global forums.

Several limitations to our study should be acknowledged. First, we have presented the responses of the coordinator from the Western Pacific Region separately rather than integrated into the primary analysis. This separation allowed methodological consistency in the analysis, while providing an indication of the situation in the Western Pacific Region. Our thematic analysis therefore covers five rather than six WHO regions. Second, our findings represent coordinators’ subjective perspectives rather than objective external assessment. This approach suits exploratory research on an under-studied topic, documenting how coordinators experience and interpret their work. However, coordinator accounts may differ from the perspectives of Member States, the WHO secretariat or documentary evidence. Future research could seek and integrate the views of multiple stakeholders. Third, purposive and snowball sampling may overrepresent coordinators with stronger networks or participation willingness, potentially missing other perspectives. Finally, protecting anonymity in small professional cohorts (typically one coordinator per region annually) is challenging. The potential for being identified may have influenced participants’ candour on politically sensitive topics, although we cannot assess the extent of this effect.

Drawing on our study findings, three policy recommendations emerge. First, WHO should establish a structured induction programme for incoming regional coordinators, delivered jointly by the Secretariat’s Governing Bodies support unit and outgoing coordinators. Such a course should cover governing body cycles, negotiation processes and communication protocols, and would address the universal finding that coordinators currently learn their role informally and often too late in their mandate. Second, a cross-regional mentorship pairing system should be instituted, matching incoming coordinators with experienced counterparts from other regions for the first 3 months of their term. Given that all our participants identified knowledge transfer as an important gap, such structured peer learning would systematize what currently depends on individual initiative. Third, WHO regional offices should develop and maintain a harmonized procedural handbook for each region covering region-specific coordination practices, key institutional contacts, governance cycles, nomination procedures and lessons learnt from previous mandates. The handbook should be updated by each outgoing coordinator. The existing handover report system of the African Region provides a proven model for adaptation across all regions. These recommendations address the structural fragility of the institutional knowledge documented in our findings and are consistent with WHO’s ongoing governance reform process.

Future research should investigate why substantial variation exists in the interpretation of the coordinator’s role across regions, include all six regions, and examine how factors such as global crises, gender, language and culture shape coordination styles and regional influence in global health governance.
